# Safety and efficacy of laronidase in Chinese patients with mucopolysaccharidosis type I: a phase IV, single-arm, open-label, multicenter study

**DOI:** 10.1186/s13023-025-04056-w

**Published:** 2025-10-29

**Authors:** Yan Liang, Yan-Ling Yang, Chao-Chun Zou, Li Liu, Ying Jiao, Yu Wang, Xin Liu, Xiao-Ping Luo

**Affiliations:** 1 Department of Pediatrics, Tongji Hospital, Tongji Medical College, Huazhong University of Science and Technology, Wuhan, 430030 China; 2 Hubei Key Laboratory of Pediatric Genetic Metabolic and Endocrine Rare Diseases, Wuhan, 430030 China; 3 Department of Pediatrics, Peking University First Hospital, Beijing, 100034 China; 4 Department of Endocrinology, Children’s Hospital of Zhejiang University School of Medicine, Hangzhou, 310003 China; 5 Department of Genetics and Endocrinology, Guangzhou Women and Children’s Medical Center, Guangzhou Medical University, Guangzhou, 510623 China; 6 Rare Disease & Rare Blood Disease, Research and Development China, Sanofi (China) Investment Co., Ltd., Beijing, 100022 China; 7 Rare Disease & Rare Blood Disease, Medical Department, Sanofi (China) Investment Co., Ltd., Shanghai, 200040 China; 8 Present Address: Medical Department, Boehringer Ingelheim (China) Investment Co., Ltd, Shanghai, 200040 China

**Keywords:** Mucopolysaccharidosis type I, Enzyme replacement therapy, Laronidase, Phase IV study, Chinese

## Abstract

**Background:**

Mucopolysaccharidosis type I (MPS I) is a lysosomal storage disorder caused by deficiency of the enzyme α-L-iduronidase. Laronidase (Aldurazyme^®^) stands as the sole FDA-approved enzyme replacement therapy (ERT) for MPS I to date. In June 2020, a concentrated solution of laronidase for injection received approval for a Chinese bioproduct license, exempted from clinical trials. Compliance with approval requirements mandates post-marketing surveillance (PMS) for laronidase. The objective of this study was to evaluate the safety and efficacy of laronidase treatment at a dosage of 100 U/kg body weight weekly in Chinese patients with MPS I.

**Methods:**

From October 2021 to July 2023, 12 MPS I patients at four institutions in China received weekly intravenous injections of laronidase at a dose of 100 U/kg of body weight once a week for 26 weeks. The primary efficacy endpoint was the percentage change in urinary glycosaminoglycans (uGAGs) levels at week 26 relative to baseline. Safety endpoints included the incidence of adverse events (AEs), serious adverse events (SAEs), and adverse events of special interest (AESIs, including infusion-related reactions) during the treatment period (TE).

**Results:**

Laronidase consistently reduced uGAGs levels from baseline to week 26, with a percentage change of -64.61% ± 26.90% (95% CI: -81.70% to -47.52%). There was a revealed reduction following laronidase treatment. The percentage reductions in uGAGs from baseline to weeks 2, 4, 8, 12, and 20 were decreased. The decreases in absolute change of uGAGs were observed at weeks 2, 4, 8, 12, 20, and 26. The percentage reduction in liver volume from baseline to week 26 was − 13.24% ± 7.86% (95% CI: -18.24% to -8.25%). Nine participants (75%) achieved an overall treatment compliance rate ≥80%. Eleven participants (91.7%) experienced treatment-emergent adverse events (TEAEs). Four participants (33.3%) experienced AESIs.

**Conclusions:**

In Chinese patients with MPS I, laronidase as an enzyme replacement therapy effectively reduces glycosaminoglycan storage and liver volume while demonstrating a favorable safety profile.

**Clinical trial registration number:**

NCT05134571.

**Clinical trial registration date:**

2021-10-21.

**Name of the registry:**

China Post-marketing Surveillance (PMS) Study of Aldurazyme^®^.

**URL of the trial registry record:**

https://clinicaltrials.gov/search?term=NCT05134571

## Introduction

Mucopolysaccharidosis type I (MPS I) is caused by mutations in the gene encoding α-L-iduronidase (IDUA). Those mutations lead to a deficiency or reduction of IDUA enzyme activity within lysosomes, which results in incomplete degradation and subsequent accumulation of mucopolysaccharides, also known as glycosaminoglycans (GAGs), within the body. This accumulation impairs the normal function of affected tissues and organs [1, 2]. The global incidence rate of MPS I ranges from 0.69 to 1.66 per 100,000 individuals [3]. In East and South China, MPS I accounts for 16% and 12% of all MPS cases, respectively [4, 5]. MPS I can be categorized based on disease severity into severe (also known as classic, Hurler syndrome), intermediate (Hurler-Scheie syndrome), and mild (Scheie syndrome) subtypes; the latter two are collectively referred to as attenuated MPS I [6]. Regardless of the subtype, MPS I follows a chronic and progressive disease course that affects multiple organs [7]. The median age of diagnosis for children with MPS I is typically around 10 months. Without timely intervention, most affected children do not survive beyond their tenth birthday. Early diagnosis and prompt treatment are of paramount importance in improving the long-term prognosis of children with MPS I [8].

Current treatments for MPS I encompass hematopoietic stem cell transplantation (HSCT) and enzyme replacement therapy (ERT). HSCT is typically the primary choice for severe cases in children aged ≤2.5 years. For children aged >2.5 years with severe or attenuated forms, HSCT may also be considered after a multidisciplinary evaluation of the transplantation risks and benefits. Alternatively, recombinant human α-L-iduronidase (administered as laronidase, marketed as Aldurazyme^®^) can be chosen for ERT [2], which aims to restore sufficient enzyme activity to hydrolyze stored GAGs and prevent their further accumulation in tissues [9]. Laronidase holds the distinction of being the world’s first and currently the only approved ERT drug for treating non-neurological manifestations of MPS I, having received approval in 2003 [10]. A phase III clinical study and a 3.5-year open-label extension study have demonstrated that laronidase significantly enhances respiratory function and physical fitness in MPS I patients, markedly reduces hepatomegaly and GAG storage, and has a commendable safety profile [11, 12]. Long-term outcome studies corroborate that the currently approved dose of laronidase (100 U/kg body weight weekly) consistently provides clinical benefits to patients with MPS I, with these benefits typically be maintained over prolonged treatment periods [13].

Laronidase, a biological product, has been primarily associated with mild to moderate infusion-related reactions (IARs) in clinical trials. In a phase III study involving 45 patients aged 6 to 43 years, 53% reported experiencing IARs during laronidase treatment over a maximum duration of 4 years [11, 12]. Similarly, a phase II study involving 20 patients under 5 years of age found that seven (35%) patients experienced 33 IARs over a maximum treatment duration of 1 year [14]. Beyond the IARs documented in clinical trials, there have been reports of severe, life-threatening IARs, including anaphylactic shock and laryngeal edema, in patients treated with laronidase [15]. Most IARs can be effectively managed by reducing the infusion rate and/or administering pre-treatment with antipyretics and/or antihistamines [6].

In June 2020, laronidase for injection was granted approval for a Chinese biological products license through the Clinical Trial Waiver. Therefore, this study aims to firstly evaluate the safety and efficacy of laronidase treatment at a dosage of 100 U/kg body weight weekly in Chinese patients with MPS I.

## Methods

### Study design

**Fig. 1:**
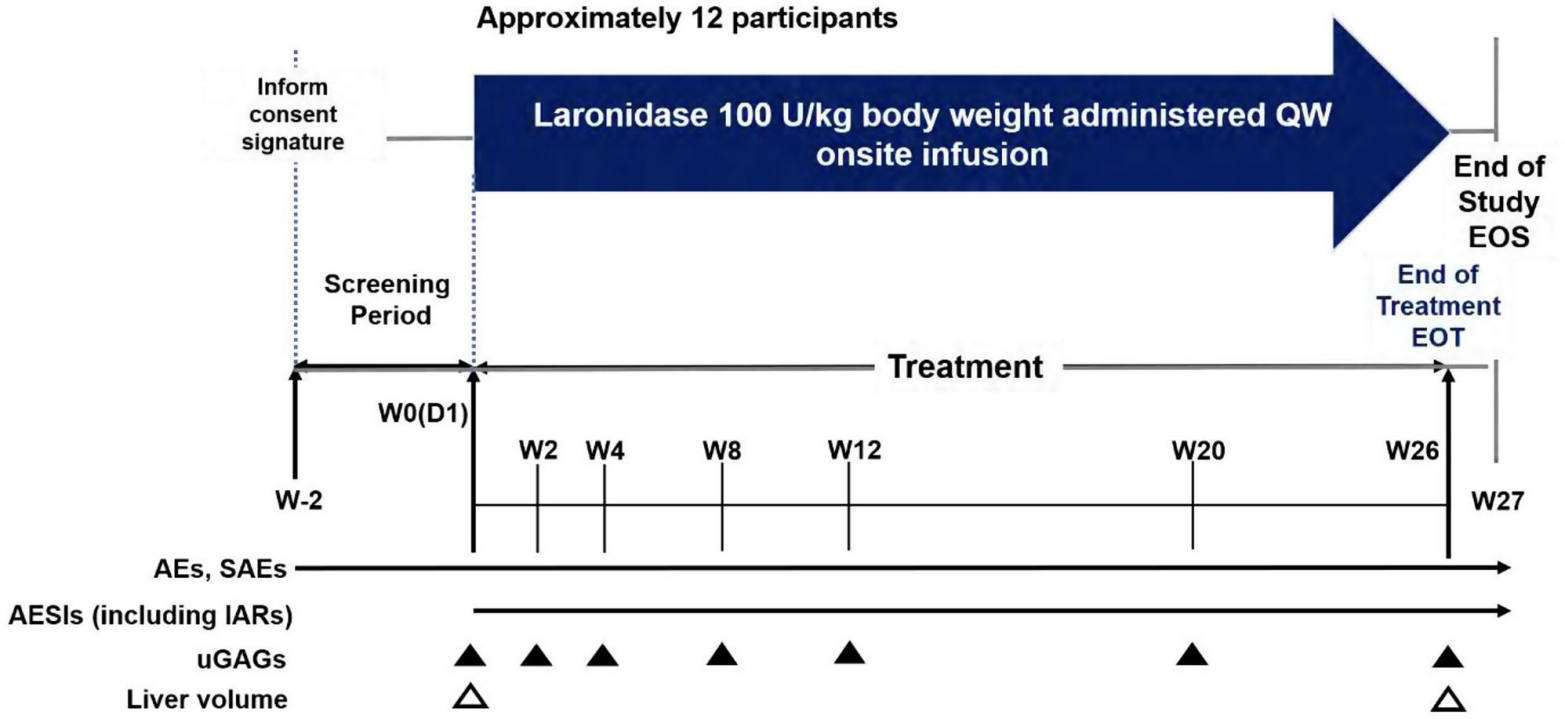
Graphical Study Design. AE, adverse event; AESI, adverse event of special interest; D, day; EOS. end of study; EOT, end of treatment; IAR, infusion associated reaction; QW, every week; SAE, serious adverse event; uGAG, urinary glycosaminoglycan; W, week

This phase IV, multicenter, open-label, single-arm, and post-marketing surveillance (PMS) study (NCT05134571) was conducted in Chinese patients with MPS I. The study initiation date was defined as the day clinical recruitment commenced. The total study duration for each subject was 29 weeks, including a 2-week screening period, a 26-week treatment period, and a 1-week follow-up period. Participants who completed all study phases, including the final visit at the end of Week 28 of treatment, were considered to have completed the clinical trial (Fig. 1).

### Study population

This study was continuously conducted from October 2021 to July 2023 across four institutions in China: Tongji Hospital Affiliated to Tongji Medical College of Huazhong University of Science and Technology, Peking University First Hospital, Children’s Hospital Affiliated to Zhejiang University School of Medicine, and Women and Children’s Medical Center Affiliated to Guangzhou Medical University. A total of 12 patients diagnosed with MPS I were enrolled. Key inclusion criteria: (1) subjects aged ≥5 years at the time of informed consent; (2) a documented diagnosis of MPS I confirmed by measurable clinical signs and symptoms, as well as fibroblast or leukocyte IDUA enzyme activity <10% of normal values; (3) subjects capable of standing independently and walking a minimum of 5 meters within 6 min; (4) informed consent signed by the subjects. Key exclusion criteria: (1) participants with a history of tracheostomy, bone marrow transplantation, or hematopoietic stem cell transplantation; (2) plans to undergo bone marrow transplantation or hematopoietic stem cell transplantation within half a year post-enrollment; (3) receipt of any investigational drug or device, other than laronidase, within 30 days before study enrollment; (4) prior experimental gene therapy; (5) pregnant or lactating women; (6) those deemed unsuitable for participation by the investigator due to medical or clinical conditions or potential non-compliance with study procedures; (7) allergy to any study intervention or its components, or any other contraindications for study participation as determined by the investigators.

### ERT medication

Laronidase was administered via intravenous injection once a week at a dose of 100 U/kg body weight. The concentrated solution of laronidase was diluted with a 0.9% sodium chloride infusion solution and prepared as a 100 mL infusion for participants weighing ≤20 kg or a 250 mL infusion for those weighing >20 kg. The total infusion duration ranged from approximately 3 to 4 h. If the patient tolerated the treatment well, the initial infusion rate started at 2 U/kg/h, with incremental increases every 15 min up to a maximum infusion rate of 32 U/kg/h. Subsequently, the maximum rate could be further increased to 43 U/kg/h with good tolerance. In the event of a recurrent infusion-associated reaction (IAR) or a severe IAR, upon resumption of dosing, the infusion rate was reduced to 1/4 to 1/2 of the rate at which the reaction initially occurred. To minimize the risk of IARs, pre-treatment medication was administered at least 1 h prior to the infusion.

### Study endpoints

The primary efficacy endpoint was the percentage change in uGAGslevels at Week 26 relative to baseline, adjusted for the modified intention-to-treat (mITT) population. If the uGAG value at Week 26 was missing, the last observed value during the treatment period was carried forward using the Last Observation Carried Forward (LOCF) method. Secondary efficacy endpoints included percentage change in uGAGs levels relative to baseline at Week 2, 4, 8, 12, and 20, the absolute change in uGAGs levels relative to baseline at Week 2, 4, 8, 12, 20, and 26, and the percentage change in liver volume at Week 26 relative to baseline. Total sulfated uGAGs levels were determined by Blyscan Assay using a commercial the Biocolor quantification kit (Cat. No. B3000) with the dye of 1, 9-dimethylmethylene blue, and liver volume was assessed by abdominal ultrasonography. Safety endpoints included the incidence of adverse events (AEs), serious adverse events (SAEs), and adverse events of special interest (AESIs, including IARs) during the treatment period (TE). Additionally, changes in electrocardiogram (ECG) readings, clinical laboratory parameters (hematology and biochemistry), and vital signs (body temperature, heart rate, respiratory rate, blood pressure) were monitored and assessed during the TE.

### Statistical analysis

The data were subjected to descriptive analysis. Continuous variables were summarized using the following statistics, count (N), mean, standard deviation (SD), minimum value, median, 1st quartile, 3rd quartile, and maximum values. In addition, for average absolute change or average percentage change from baseline, 95% confidence intervals (CI) were calculated. Categorical variables were summarized using frequencies and percentages. For binary endpoints, 95% CIs were also calculated.

## Results

### Demographic data

A total of 12 participants were screened and enrolled in this study, including 8 (66.7%) males and 4 (33.3%) females, all of them were Asian (Table 1). All participants had attenuated forms of MPS I. Their average age was 13.8 ± 7.7 years, ranging from 6 to 28 years. Among them, 7 (58.3%) were aged 5 to 11 years, 1 (8.3%) were aged 12 to 17 years, and 4 (33.3%) were aged more than 18 years. Their average body weight was 45.7 ± 11.9 kg for adults and 23.6 ± 7.5 kg for pediatrics. The average height for adults and pediatrics was 142.3 ± 9.8 cm and 109.1 ± 15.6 cm, respectively. A total of 11 (91.7%) participants had more than 1 medical or surgical history.

**Table 1:** Demographic Characteristics at Baseline for the Enrolled Population

Subject No.	Age	Gender	Date of First Exposure to Treatment	Date of Last Exposure to Treatment	Alpha-L-Iduronidase (nmol/hr/mg protein)						Urine Glycosaminoglycan (mg/L)			
					Baseline Value	Analysis Normal Range Lower Limit	Analysis Normal Range Upper Limit	Date/Time of Specimen Collection	Analysis Age at Visit	Specimen Type	Baseline Value	Date/Time of Specimen Collection	Analysis Age at Visit	Specimen Type
1	19	M	2021-11-11	2022-05-10	1.4	27.2	52.0	2011-06-21	9.02	leucocytes	520.9	2021-11-10	19.41	urine
2	8	M	2022-05-25	2022-11-23	1.39	21	—	2015-06-01	0.96	leucocytes	372.7	2022-05-24	7.94	urine
3	11	F	2022-08-24	2023-02-24	0.59	18.70	156.10	2012-11-13	2.42	leucocytes	946.3	2022-08-23	12.19	urine
4	11	M	2022-10-12	2023-04-14	1.8	27.2	52.0	2016-01-10	4.57	leucocytes	276.2	2022-10-11	11.33	urine
5	7	M	2022-04-07	2022-10-09	0.22	12.17	277.51	2016-07-04	1.06	leucocytes	340.4	2022-04-07	6.81	urine
6	6	M	2022-05-05	2022-11-02	0.30	12.17	277.51	2018-09-02	3.22	leucocytes	570.3	2022-05-05	6.89	urine
7	28	F	2022-12-09	2023-06-08	0.0	19.6	30.6	1997-10-31	4.38	leucocytes	388.3	2022-12-09	29.49	urine
8	7	M	2022-07-29	2023-02-03	0.0	25.4	118.5	2016-03-22	0.77	leucocytes	350.2	2022-07-29	7.12	urine
9	12	F	2022-08-08	2023-02-07	0.0	25.4	118.5	2016-03-22	6.77	leucocytes	327.7	2022-08-08	13.15	urine
10	21	M	2022-08-26	2023-02-24	0.0	12.17	277.51	2013-07-31	12.13	leucocytes	292.7	2022-08-26	21.20	urine
11	10	M	2022-09-27	2023-03-31	0.83	12.17	277.51	2016-02-23	3.69	leucocytes	389.2	2022-09-27	10.29	urine
12	26	F	2023-01-18	2023-07-21	3.93	21	—	2022-11-17	26.43	fibroblast	310.2	2023-01-18	26.60	urine

M: male; F: female

### Laronidase exposure

All participants received at least 1 dose of the study intervention. The cumulative treatment exposure was 6.25 participant-years, with an average treatment duration of 27.19 ± 0.35 weeks (Table 2). All subjects were exposed to the study intervention for more than 13 weeks, and no significant impact of COVID-19 on the study intervention exposure was noticed.

**Table 2:** Extent of Exposure to Investigational Medicinal Products for the Safety Population

Items	Laronidase [*n*(%)]
Cumulative duration to treatment exposure (participant years)	6.25
Duration of IMP exposure (weeks)	
Number	12
Mean ± SD	27.19 ± 0.35
Duration of IMP exposure by category	
Missing duration	0
>1 week and ≤13 weeks	0
>13 weeks	12 (100)
Cumulative duration of treatment exposure by category	
≥1 week	12 (100)
≥13 weeks	12 (100)

IMP: Investigational medicinal product; Percentages are calculated using the number of participants in the safety population with a non-missing duration of exposure as denominator; Duration of IMP exposure is defined as last IMP administration date - first IMP administration date + 7 days, regardless of unplanned intermittent discontinuations

### Efficacy endpoints

All efficacy analyses were conducted on the mITT population. All participants had evaluable uGAGs at baseline and week 26. The average percent change of uGAGs from baseline to Week 26 was −64.61% ± 26.90%, with a 95% CI of -81.70% to -47.52% (Table 3). The average percentage reduction of uGAGs from baseline to Weeks 2, 4, 8, 12, and 20 were −32.23% ± 29.37%, -55.21% ± 30.86%, -59.79% ± 22.48%, -53.15% ± 23.48%, -60.15% ± 27.16%, respectively (Table 4).

**Table 3:** Percent Change of uGAGs from Baseline to Week 26 for the mITT Population

Items	Baseline (mg/L)	Week 26 (mg/L)	Percent change from baseline
Number	12	12	
Mean ± SD	423.76 ± 186.46	125.96 ± 74.19	-64.61 ± 26.90
Median	361.45	123.35	-70.56
Q1 ; Q3	318.95 ; 455.05	62.10 ; 163.60	-81.51 ; -54.90
Min ; Max	276.2 ; 946.3	35.4 ; 276.7	-95.6 ; 0.2

uGAG: urinary glycosaminoglycan; For the uGAG value at Week 26 that is missing, the missing value is imputed by carrying forward the last uGAG value (LOCF method, last observation carried forward) observed during the on-treatment period

**Table 4:** Percent Change of uGAGs from Baseline up to Week 20 for the mITT Population

Items	Number	Mean ± SD	Median	Q1 ; Q3	Min ; Max	95% CI
Baseline (mg/L)	12	423.76 ± 186.46	361.45	318.95 ; 455.05	276.2 ; 946.3	
Week 2 (mg/L)	10	292.04 ± 144.46	302.20	183.40 ; 378.30	96.6 ; 564.1	
Percent change from baseline	10	-32.23 ± 29.37	-40.47	-52.77 ; 1.48	-67.0 ; 14.5	(-53.24 ; -11.22)
Week 4 (mg/L)	10	185.38 ± 122.33	160.25	78.20 ; 286.20	49.3 ; 420.6	
Percent change from baseline	10	-55.21 ± 30.86	-59.51	-71.69 ; -48.00	-84.7 ; 23.6	(-77.29 ; -33.14)
Week 8 (mg/L)	10	145.33 ± 86.20	105.70	97.90 ; 250.80	27.8 ; 273.1	
Percent change from baseline	10	-59.79 ± 22.48	-65.11	-74.56 ; -48.16	-91.8 ; -21.2	(-75.88 ; -43.71)
Week 12 (mg/L)	11	183.38 ± 73.39	191.50	121.40 ; 231.60	56.4 ; 304.6	
Percent change from baseline	11	-53.15 ± 23.48	-58.52	-75.19 ; -35.84	-85.5 ; -10.5	(-68.92 ; -37.38)
Week 20 (mg/L)	11	162.72 ± 104.49	120.40	80.20 ; 243.30	41.6 ; 379.7	
Percent change from baseline	11	-60.15 ± 27.16	-71.25	-79.35 ; -46.15	-85.8 ; 1.9	(-78.40 ; -41.91)

uGAG: urinary glycosaminoglycan

Continuous decreases in the average absolute change of uGAGs were observed from baseline up to Week 26. The average absolute changes at Weeks 2, 4, 8, 12, 20, and 26 were −149.93 ± 138.55 mg/L, -240.02 ± 181.58 mg/L, -216.46 ± 92.59 mg/L, -250.70 ± 194.64 mg/L, -269.77 ± 199.82 mg/L, -297.80 ± 225.55 mg/L, respectively (Table 5).

**Table 5:** Absolute Change of uGAGs (mg/L) from Baseline up to Week 26 for the mITT Population

Items	Number	Mean ± SD	Median	Q1 ; Q3	Min ; Max	95% CI
Baseline	12	423.76 ± 186.46	361.45	318.95 ; 455.05	276.2 ; 946.3	
Week 2	10	292.04 ± 144.46	302.20	183.40 ; 378.30	96.6 ; 564.1	
Absolute change from baseline	10	-149.93 ± 138.55	-194.05	-209.10 ; 5.50	-382.2 ; 49.5	(-249.05 ; -50.81)
Week 4	10	185.38 ± 122.33	160.25	78.20 ; 286.20	49.3 ; 420.6	
Absolute change from baseline	10	-240.02 ± 181.58	-220.70	-246.50 ; -194.10	-660.1 ; 80.2	(-369.92 ; -110.12)
Week 8	10	145.33 ± 86.20	105.70	97.90 ; 250.80	27.8 ; 273.1	
Absolute change from baseline	10	-216.46 ± 92.59	-215.75	-312.60 ; -149.40	-319.5 ; -69.5	(-282.70 ; -150.22)
Week 12	11	183.38 ± 73.39	191.50	121.40 ; 231.60	56.4 ; 304.6	
Absolute change from baseline	11	-250.70 ± 194.64	-197.70	-331.90 ; -99.00	-714.7 ; -35.8	(-381.46 ; -119.94)
Week 20	11	162.72 ± 104.49	120.40	80.20 ; 243.30	41.6 ; 379.7	
Absolute change from baseline	11	-269.77 ± 199.82	-251.10	-308.10 ; -161.60	-774.8 ; 7.0	(-404.02 ; -135.53)
Week 26	12	125.96 ± 74.19	123.35	62.10 ; 163.60	35.4 ; 276.7	
Absolute change from baseline	12	-297.80 ± 225.55	-246.35	-360.80 ; -182.65	-904.8 ; 0.5	(-441.10 ; -154.50)

uGAG: urinary glycosaminoglycan; For the uGAG value at Week 26 that is missing, the missing value is imputed by carrying forward the last uGAG value (LOCF method, last observation carried forward) observed during the on-treatment period

**Fig. 2:**
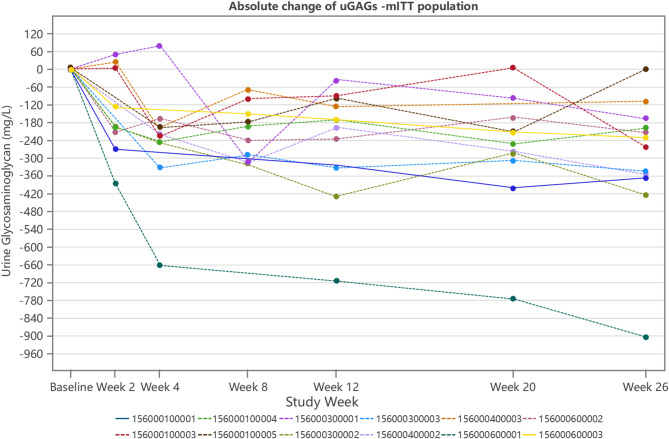
Spaghetti Plot Absolute Change of uGAGs - mITT Population. uGAG, urinary glycosaminoglycan

Individual subject data regarding the absolute change in uGAGs from baseline up to Week 26 were displayed in Fig. 2. All participants demonstrated reductions in uGAGs from baseline to Week 26, except for 1 pediatric participant (Participant No. LPS16578-156-0001-00005), who experienced a slight uGAG increase (0.5 mg/L) at Week 26 in comparison with the baseline. However, it’s worth noting that this participant had significantly decreased uGAGs levels (a minimum decrease of >60 mg/L) after receiving laronidase administration at prior treatment weeks (Fig. 2; Table 5). The average percentage reduction of liver volume from baseline to Week 26 was − 13.24% ± 7.86% (Table 6).

**Table 6:** Percent Change of Liver Volume (cc) from Baseline to Week 26 for the mITT Population

Items	Baseline	Week 26	Percent Change from Baseline
Number	12	12	12
Mean ± SD	817.22 ± 169.14	707.02 ± 152.40	-13.24 ± 7.86
Median	786.07	658.62	-15.76
Q1 ; Q3	684.01 ; 959.29	611.63 ; 788.80	-18.69 ; -6.03
Min ; Max	580.3 ; 1123.9	471.8 ; 1014.9	-25.1 ; 0.6
95% CI			(-18.24 ; -8.25)

Liver volume is measured by Abdominal B type Ultrasound examination

### Safety endpoints

Despite the COVID-19 pandemic that occurred during the study, the average percentage of treatment compliance was 90.12% ± 10.03%. Nine(75.0%) participants had an overall treatment compliance rate of ≥80%. Eleven(91.7%) participants experienced TEAEs during the treatment, with 1(8.3%) having a treatment-emergent serious AE (hypertension, mild and not laronidase-related per investigator’s assessment). The majority of the TEAEs were mild to moderate in intensity. Commonly reported TEAEs by participants were upper respiratory tract infection and cough 3(25.0%) participants each; all events were assessed as not related to laronidase). Treatment-related TEAEs were reported in 5(41.7%) participants, one each of hypotension, erythematous rash, urticaria, and fical oedema. and blood pressure increased. No participants died or experienced TEAEs leading to permanent treatment discontinuation. Four (33.3%) participants experienced adverse events of special interest (AESIs), all of which were IARs. There were no clinically significant changes observed over time in clinical laboratory parameters (hematology, liver function, metabolism, and renal functionparameters), vital signs, or ECGs.

## Discussion

This phase IV, multicenter, open-label, and single-arm PMS study was conducted in 12 Chinese patients with MPS I, representing the largest sample size to date and the only PMS study of laronidase in China. Among these 12 MPS I patients, the majority were male (66.7% vs. 33.3%). The cohort was relatively young, with 66.7% being children, and over half (58.3%) of the participants were in the 5 to 11 years age group. Chinese MPS I patients often experience significant growth and development delays, with body weight and height falling below the normal ranges for their age. MPS I involves multiple system organs, resulting in a broad spectrum of clinical manifestations and typically follows a progressive course.

ERT with laronidase has been reported to improve quality of life, including daily activity levels, exercise endurance, and overall health status [16–20]. In a phase III clinical trial and its subsequent open-label extension study, all MPS I patients were randomized to receive either laronidase at a dose of 100 U/kg (0.58 mg/kg) body weight weekly or placebo [11, 12]. After 26 weeks of treatment, laronidase significantly and consistently reduced average uGAGs levels compared to the placebo group (a decrease of 54.1% in laronidase group vs. an increase of 47.3% in the placebo group). Furthermore, there was a clinically significant improvement in forced vital capacity (FVC) with laronidase treatment (41% vs. 9%), and a reduction in liver volume to within the normal range (72% vs. 21%) [11]. A previous study conducted at St. Mary’s Hospital in Manchester, UK, tracked the effects of ERT in 35 patients with attenuated MPS I [13]. Regardless of the age at which laronidase therapy was initiated, after 6 months of treatment, the average uGAGs levels decreased by over 50% compared to baseline, and this reduction was consistently maintained throughout treatment, with reductions ranging from 50% to 90% compared to baseline levels [13]. Our phase IV study demonstrated that laronidase consistently reduced uGAGs levels from baseline throughout the treatment period, up to Week 26, with a percentage change of -64.61%. These reductions are consistent with the findings of aforementioned studies. Analyses of the secondary efficacy endpoint also indicated decreases following treatment with laronidase, including the percentage changes and absolute changes of uGAGs levels from baseline to Week 20 and 26, as well as the changes in liver volume from baseline to Week 26. In this study, uGAG was analyzed in a qualified central lab using verified method, however, the urine samples come from random urine, which may explain the fluctuated uGAGs levels at post-treatment (Fig. 2; Table 4). However, the overall trend generally shows a decrease of uGAGs from baseline to after laronidase treatment, which is consistent with global pivotal study [11]. These results further underscore the clinical benefit of laronidase in Chinese MPS I participants.

Numerous studies with long-term outcomes have substantiated the sustained efficacy of laronidase in the treatment of MPS I. A 3.5-year extension study of a phase III clinical trial demonstrated that uGAGs levels were consistently maintained at lower levels. Notably, 18% of patients exhibited clinically significant improvements in lung function (Forced Vital Capacity, FVC), while 55% maintained stability. Moreover, 50% of patients showed clinically meaningful improvements in endurance during the 6-minute walk test (6MWT), with 28% maintaining stability. Additionally, improvements were observed in joint mobility assessments, including shoulder, knee flexion, and extension. Remarkably, 92% of patients experienced a reduction in liver volume within the normal range, and growth rates(median height) in pediatric patients returned to normal. Laronidase treatment was associated with improvements in activities of daily living [12]. In addition, a key observation from the phase III open-label extension study is that various aspects of MPS I respond differentially to ERT and exhibit varying response timelines. The timing of these responses (months for urinary glycosaminoglycan, liver volume, and sleep apnea; years for endurance, mobility, and activities of daily living) plays a crucial role in guiding treatment expectations [12]. Results from a 10-year follow-up study of 35 individuals with attenuated MPS I at St. Mary’s Hospital in Manchester, UK, revealed statistically significant reductions in urinary glycosaminoglycan levels relative to baseline, observed as early as 6 months after treatment initiation and sustained throughout the follow-up period [13]. Disease stability was evident after the initiation of treatment, without statistically significant changes in average FVC, 6MWT, or height-for-age Z score. Additionally, mitral and aortic valve function remained stable in 65% (22/34) of patients, corneal clouding showed stability in 78% (18/23), and visual acuity remained stable in 33% (8/24) while improving in 42% (10/24) [13]. Long-term outcomes of Taiwanese MPS I patients demonstrated significant improvements in both biochemical and clinical parameters in all patients after 2.0-8.3 years of ERT. Laronidase led to reduced uGAGs, dermatan sulfate (DS), and heparan sulfate (HS) levels, along with improvements or stabilization in endurance, mobility, joint function, pulmonary function, liver size, and spleen size. Laronidase also appeared to effectively reduce cardiac hypertrophy, with potentially better results when treatment was initiated at a younger age [21].

HSCT is widely regarded as the gold standard for treating severe MPS I and is ideally performed early, preferably by 2.5 years of age and before the onset of cognitive impairment (developmental quotient/IQ of >70) [2, 6]. HSCT has demonstrated efficacy in improving various clinical manifestations in MPS patients, including reduced joint mobility, coarse facial features, upper airway obstruction, and hepatosplenomegaly [22]. It also significantly reduces central nervous system complications such as cervical cord compression or severe hydrocephalus [23]. However, long-term clinical outcomes can vary widely, and a persistent residual disease burden often remains [24]. The high cost of laronidase ERT imposes a significant financial burden on patients. In China, monotherapy with laronidase is less commonly administered to severe MPS I patients aged ≤2.5 years; instead, many opt for a combination of ERT and HSCT. Reports indicate that combined laronidase ERT and HSCT therapy is reported feasible and well-tolerated in pediatric patients with severe MPS I [25]. Pre-transplant ERT has been shown to effectively improve cardiopulmonary function, increase transplant success rates, and reduce complications [26–28]. Post-transplant ERT effectively lowers uGAGs levels, improves somatic outcomes and clinical prognosis, and increases survival rate [29–32]. In addition, patients, regardless of transplant success, can benefit from long-term post-transplant ERT [33]. Consequently, the combined laronidase ERT and HSCT regimen has become the standard treatment protocol in multiple medical centers [26, 32]. There have been documented successful cases of laronidase combined with HSCT in Asian countries, including China and Japan [34, 35].

Both clinical trials and long-term PMS studies have consistently affirmed the good safety and tolerability profile of laronidase. Most adverse events reported in clinical trials have been categorized as minor IARs. In a previous phase III study (with a treatment duration of up to 4 years), mild IARs were observed in 53% of patients [12], and in a study involving children under 5 years of age (with a treatment duration of up to 1 year), such reactions were observed in 35% of patients [14]. Long-term safety data after market approval indicate that while some IARs may be severe, their frequency decreases with extended treatment duration, rendering TRAEs controllable and manageable. In this phase IV study, 11 out of 12 participants (91.7%) experienced TEAEs, with only 1 (8.3%) participant experiencing a treatment-emergent serious AE (mild hypertension, not laronidase-related per investigator’s assessment). No participants experienced mortality or TEAEs that resulted in permanent treatment discontinuation. Overall, the safety outcomes in this study align with the well-established safety profile of laronidase in the MPS I population, consistent with findings from clinical trials and post-marketing experience. Notably, no new safety concerns were identified.

This study has certain limitations. Due to the rarity of MPS I, the number of cases included in this study was limited, and the study did not encompass children under 5 years of age or included a control group for comparison. MPS I is a chronic progressive disease, and different age groups may exhibit varying response times to different treatment indicators. Both the long-term safety and efficacy of laronidase need to be further investigated and explored in the real world due to the study design with only a 26-week treatment period. In addition, this study primarily focuses on safety and substrate storage indicators (uGAGs levels and liver volume). Long-term safety and benefits for other clinical endpoints (such as sleep apnea, pulmonary function, endurance, joint mobility, vision, etc.) of laronidase treatment still require further assessment in future studies. Moreover, anti-drug antibody (ADA) testing was not performed in this study, as it was not included in the original protocol. ADA formation may potentially affect the safety of enzyme replacement therapy; therefore, future studies should consider monitoring ADA levels.

## Conclusions

This study is the first to assess the safety and efficacy of laronidase ERT in Chinese patients with MPS I. Our results indicate that after 26 weeks of ERT, laronidase effectively reduced the uGAGs levels and liver volume while maintaining a favorable safety profile in all 12 patients. No new safety signals were identified. Future studies are warranted to evaluate the long-term safety and efficacy of laronidase in Chinese MPS I patients.

## Data Availability

The datasets used and analyzed during the current study are available from the corresponding author on reasonable request.

## Abbreviations

MPS I: Mucopolysaccharidosis type I
ERT: Enzyme replacement therapy
PMS: Post-marketing surveillance
FDA: Food and Drug Administration
uGAG: Urinary glycosaminoglycan
AEs: Adverse events
SAEs: Serious adverse events
AESIs: Adverse events of special interest
IARs: Infusion-associated reactions
HSCT: Hematopoietic stem cell transplantation
mITT: Modified intention-to-treat
CI: Confidence interval
ECG: Electrocardiogram
TEAEs: Treatment-emergent adverse events
FVC: Forced vital capacity
6MWT: 6-minute walk test
DS: Dermatan sulfate
HS: Heparan sulfate
